# Host traits and environmental variation shape gut microbiota diversity in wild threespine stickleback

**DOI:** 10.1186/s42523-025-00404-0

**Published:** 2025-06-18

**Authors:** Andreas Härer, Emma Kurstjens, Diana J. Rennison

**Affiliations:** 1 Department of Ecology, Behavior, & Evolution, School of Biological Sciences, University of California San Diego, La Jolla, CA USA; 2 Department of Medicine, School of Medicine, University of California San Diego, La Jolla, CA USA

**Keywords:** Gut microbiome, 16S rRNA sequencing, Animal microbiome, *Gasterosteus aculeatus*

## Abstract

**Background:**

Despite the growing recognition of the importance of gut microbiota in host ecology and evolution, our understanding of the relative contributions of host-associated and environmental factors shaping gut microbiota composition within and across wild populations remains limited. Here, we investigate how host morphology, sex, genetic divergence, and environmental characteristics influence the gut microbiota of threespine stickleback fish populations from 20 lakes on Vancouver Island, Canada.

**Results:**

Our findings reveal substantial variation in gut microbiota composition and diversity among populations, with host traits exerting a relatively stronger influence on bacterial alpha diversity than environmental characteristics. Previous studies have suggested a link between stickleback body shape and niche specialization, and our results indicate that aspects of host morphology may be associated with gut microbiota divergence among populations, though whether this is related to trophic ecology remains to be explored. Within and across populations, we only observed a weakly defined core microbiota and limited sharing of amplicon sequence variants (ASVs) among hosts, indicating that gut microbiota composition is individualized. Additionally, we detected sex-dependent differences in microbial diversity, opening avenues for future research into the mechanisms driving this variation.

**Conclusions:**

In sum, our study emphasizes the need to consider both host-associated and environmental factors in shaping gut microbiota dynamics and highlights the complex interplay between host organisms, their associated microbial communities, and the environment in natural settings. Ultimately, these insights add to our understanding of the eco-evolutionary implications of host-microbiota interactions while underscoring the need for further investigation into the underlying mechanisms.

**Supplementary Information:**

The online version contains supplementary material available at 10.1186/s42523-025-00404-0.

## Background

The gut microbiota plays a vital role in many aspects of an organism’s biology, which can have eco-evolutionary implications [27, 34, 51, 66, 77]. Understanding the dynamics that shape gut microbiota of animals is therefore of particular importance in wild populations, where organisms are exposed to complex environmental pressures and interactions often absent in controlled settings. Numerous studies across a broad range of study systems have highlighted the multifaceted influences of host traits and environmental factors on gut microbiota composition and diversity. Key host traits affecting the gut microbiota include genetics [8, 18], immune system function [14, 36], sex [43, 76], and trophic ecology [5, 23]. Important environmental factors include habitat type [72], physicochemical characteristics [9, 24], geographic context [50, 52], and interaction with other hosts [19, 40]. Studying multiple key factors within a single system and thereby disentangling their relative contributions can be challenging, but it offers the potential to reveal new insights into how gut microbial communities are structured in wild populations. Host species that have recently and repeatedly colonized distinct habitats and adapted to local conditions can offer valuable insights into the factors driving gut microbiota variation across ecologically and genetically divergent, geographically isolated natural replicates.

The threespine stickleback fish (*Gasterosteus aculeatus*, hereafter referred to as ‘stickleback’), a well-known model system for ecology and evolution [6], represents such a system. Since the end of the last ice age (10,000–12,000 years ago), marine stickleback independently colonized and adapted to thousands of freshwater habitats. Among these freshwater populations there is substantial ecological, morphological, and genetic divergence [6, 46, 62, 68, 69]. A particularly important axis of divergence is reflected in their trophic ecology. Freshwater stickleback typically consume two distinct types of prey found in different habitats; benthic prey, which are littoral invertebrates found in the sediment, and limnetic prey, which are pelagic zooplankton found in the open water of lakes [6]. Variation in the proportion of these diet types has been detected within and across lakes, and stickleback’s trophic ecology ranges from benthic specialists to generalists to limnetic specialists [12, 69]. Variation in trophic ecology is associated with stickleback morphology in terms of overall body shape, as well as, the feeding apparatus and body armor [6, 47, 68]. Given this remarkable ecological and morphological diversity, stickleback are instrumental for investigating gut microbiota dynamics and the interplay between the environment, host organisms and their associated microbial communities.

Stickleback are also emerging as a model system for gut microbiota research, and initial work has greatly advanced our understanding of how host traits and environmental factors contribute to gut microbiota variation [15, 16, 30, 31, 49, 63, 73, 75]. Previous work has indicated that host traits affecting gut microbiota composition include sex [16], genomic variation [73–75], diet and ecotype [15, 31, 63, 74]. These studies have laid an essential foundation for our understanding of the stickleback gut microbiota. However, they come with certain limitations for advancing our understanding of the factors that shape ecologically relevant gut microbiota variation within and across wild host populations. For example, previous studies were either restricted to a single or few lake populations [14–16, 49, 74, 75], included very few individuals per population hindering the assessment of within-population diversity [63], or were conducted in the lab [30, 73].

**Fig. 1:**
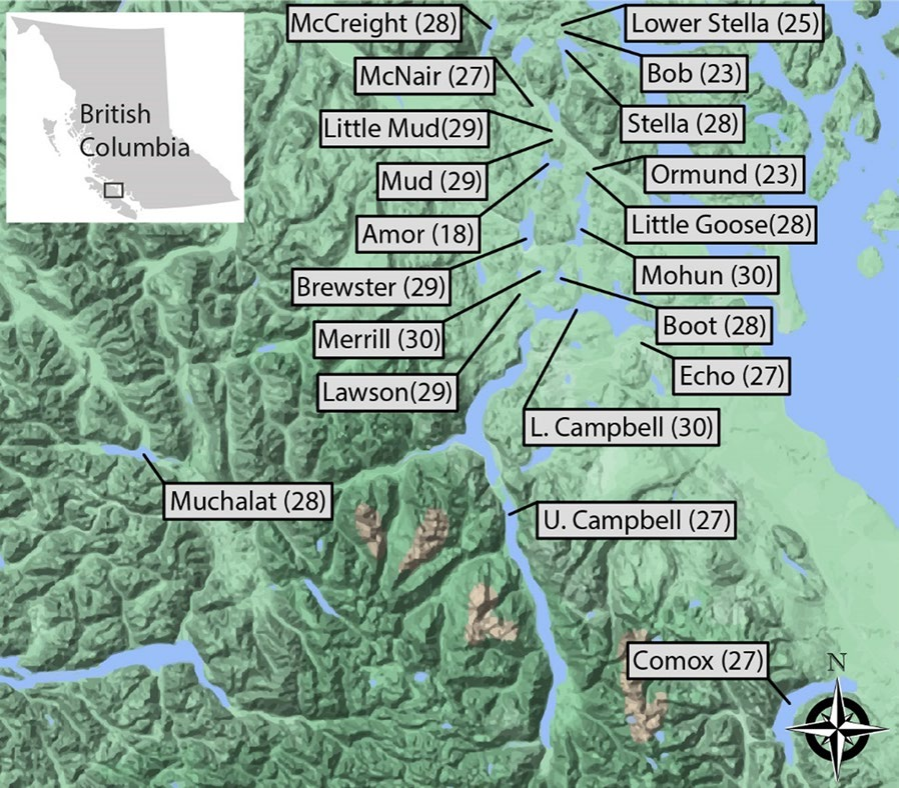
Map of Vancouver Island, Canada, showing locations of the 20 lakes included in this study. For each lake the sample size for the corresponding stickleback population is indicated. The lakes are distributed across several watersheds and this map provides an overview of the geographic distribution of the sampled populations. These data allow investigating how ecological, geographic, and environmental factors influence the stickleback gut microbiota

Building on the progress made in earlier studies, we sought to explore host-microbiota interactions more comprehensively in wild populations, with the aim of disentangling the relative contributions of host traits and environmental factors to gut microbiota composition and diversity. Specifically, we asked: (i) how key host traits, such as sex, morphology, and genetic differentiation, shape gut microbiota diversity and composition, and (ii) to what extent environmental factors, including the physicochemical characteristics of lake environments, bacterial communities in the surrounding water, and geographic distance between populations, influence gut microbiota structure. To address these questions, we surveyed stickleback populations from twenty lakes on Vancouver Island, Canada (Fig. 1).

We tested the effects of these factors on gut microbiota alpha diversity (bacterial diversity of individual hosts) and beta diversity (dissimilarity of bacterial communities among hosts) across populations, as well as the effects of host morphology and sex on alpha and beta diversity within populations. Alpha diversity is a key metric for understanding the richness and evenness of microbial communities within individual hosts, which has been shown to be associated with ecologically relevant host traits such as diet [4], growth rate [32], and survival [9]. In contrast, beta diversity captures the variation in microbial community composition among individuals, providing insights into the factors that drive gut microbiota divergence, such as ecological, genetic, or environmental influences. By examining these two complementary aspects of diversity, we aim to gain a comprehensive understanding of how gut microbiota are structured within and across populations. While the across-population analysis allowed us to evaluate overall patterns of gut microbiota diversity, the within-population analysis enabled us to identify population-specific patterns, providing insights into how these associations might vary among populations. This approach is useful given previous findings in stickleback that sexually dimorphic traits, including trophic traits, often show population-specific patterns [13]. In this study, we integrate diverse ecological and evolutionary influences into a single framework to unravel their relative contributions to gut microbiota composition and diversity. This approach provides novel insights into the structuring of the gut microbiota and the geographic distribution of microbial diversity across wild freshwater fish populations.

## Methods

### Sampling and data collection

We collected stickleback using minnow traps in the spring of 2020, 2021, and 2022 under British Columbia Fish Collection permits NA20-602264, MRVI21-619,908, and NA22-713,085, respectively. The twenty lakes examined in this study are distributed across seven watersheds. While some lakes are within the same watershed, most are not connected via streams. The exceptions are Upper and Lower Campbell, which are separated by a dam, Mohun and Little Goose, and Stella and Bob, which are connected by streams. In the field, we euthanized fish with an overdose of MS-222 (500 mg/L) and stored them at -20°C until gut dissection. In the same year of fish sampling, we further collected four water samples from the shoreline of each lake. Precautionary measures were taken to mitigate contamination risk across lakes. We sterilized sampling equipment (forceps, syringes, filter housings, and 1.5 mL tubes) using UV light before the field trip and we assembled individual sampling kits for each lake. These kits were kept sealed until sampling at the designated location. We used disposable gloves and avoided direct contact with the water to prevent human contamination. Each sample measured 120 mL and was manually filtered through a cellulose nitrate filter (Whatman plc, Maidstone, UK; ø 25 mm, pore size 0.2 µm) using a 50 mL sterile syringe equipped with a Luer lock. Following filtration, we carefully removed the filters from the housing using sterile disposable forceps and transferred them into 1.5 mL tubes. These tubes were then stored at − 20 °C until DNA extraction.

To determine body shape of all fish included in our study, we used geometric morphometric measurements of stickleback body shape based on 17 digital landmarks [2]. To collect data, we took standardized photographs of each specimen’s left lateral side including a ruler for scale. We used tpsDig2 [65] for landmarking and imported data into morphoJ [37] for Procrustes fitting and creating a covariance matrix. We then imported these files into R v4.2.1 [59] where we created Mahalanobis distance matrices on the individual and population level (*mahalanobis* function of the stats package v4.2.1) that were used for subsequent analyses. We converted the Procrustes data into a 3D array and performed PCA using the *gm.prcomp* function of the geomorph package (v4.0.7; [1]). To examine morphological variation along the major principal components, we performed a Generalized Procrustes Analysis (GPA) using the *gpagen* function and extracted the consensus shape. Using the PCA results, we defined the extreme shapes (minimum and maximum) along the first three axes and visualized the associated shape changes with the *plotRefToTarget* function. These visualizations highlighted the morphological changes associated with negative and positive scores of the first three PC axes. Body length was measured for all specimens using digital calipers. Further, we obtained pairwise F_ST_ values from Bolnick and Ballare [12] for sixteen of the populations included in our study to determine levels of genetic differentiation among populations. These F_ST_ values are based on stickleback collected in the same lakes but in different years compared to our samples. Geographic distances between lakes were measured as straight-line paths using the closest points along their shorelines, as determined with the distance measurement feature in Google Maps [25]. In the spring of 2022, we collected various physicochemical characteristics of the lakes (temperature, dissolved oxygen, conductivity, pH) using a YSI Professional Plus device (YSI Incorporated, Yellow Springs, OH). For each lake, we collected three replicate measurements and averaged the values.

### Sample processing and library preparation

In the lab, we rinsed fish with EtOH and dissected their whole guts using sterile equipment. To minimize the abundance of transient bacteria, we carefully removed gut contents by squeezing. We then stored gut samples at − 80 °C until DNA extraction. We extracted DNA from fish guts with the QIAGEN PowerSoil Pro Kit according to the manufacturer’s protocol (Qiagen, Hilden, Germany) and from cellulose nitrate filter with the QIAGEN DNeasy Blood & Tissue kit (Qiagen, Hilden, Germany) with minor modifications. As a first step, we immersed filters in 450 µl buffer ATL and 50 µl proteinase K and incubated them at 65 °C for 1 h. Then, we added 500 µl buffer AL and 500 µl 100% ethanol and rigorously vortexed the tubes. Next, we applied the mixture to a DNeasy Mini spin and performed the rest of the extraction according to the manufacturer’s protocol. DNA extractions were done under sterile conditions in a laminar flow hood.

To characterize the taxonomic composition of host-associated and free-living bacterial communities, we used a 16S rRNA gene metabarcoding approach. Specifically, we amplified the V4 region of the 16S rRNA gene with barcoded 515F and 806R primers (for information on primer and barcode sequences see https://github.com/SchlossLab/MiSeq_WetLab_SOP/blob/master/MiSeq_WetLab_SOP.md). We conducted PCR amplification in triplicate, using a 10 μl reaction volume and the Platinum II Hot Start PCR Master Mix (Thermo Fisher Scientific, Waltham, MA). Subsequently, we pooled the three replicates. We included negative controls of sterile water during DNA extraction and PCR amplification, which did not yield measurable DNA concentrations. The PCR protocol involved an initial denaturation step for 60 s at 98 °C, followed by 35 amplification cycles with 10 s at 98 °C, 20 s at 56 °C, and 60 s at 72 °C, and a final elongation step for 10 min at 72 °C. We visually confirmed amplification of fragments of expected size by gel electrophoresis (2% agarose gels) and we measured DNA concentrations of amplicons with a Qubit 4 Fluorometer (Thermo Fisher Scientific, Waltham, MA). Finally, we pooled samples in an equimolar manner, and the final libraries were sequenced on the Illumina MiSeq 600 (PE300) platform at the UC Davis Genome Center after bead clean-up and quality check on a Bioanalzyer (Agilent Technologies, Santa Clara, CA). Unfortunately, DNA extraction and amplification failed for water samples from Ormund Lake, leaving us with nineteen lakes for which we have information for host-associated and free-living bacterial communities.

### Data analysis

**Fig. 2:**
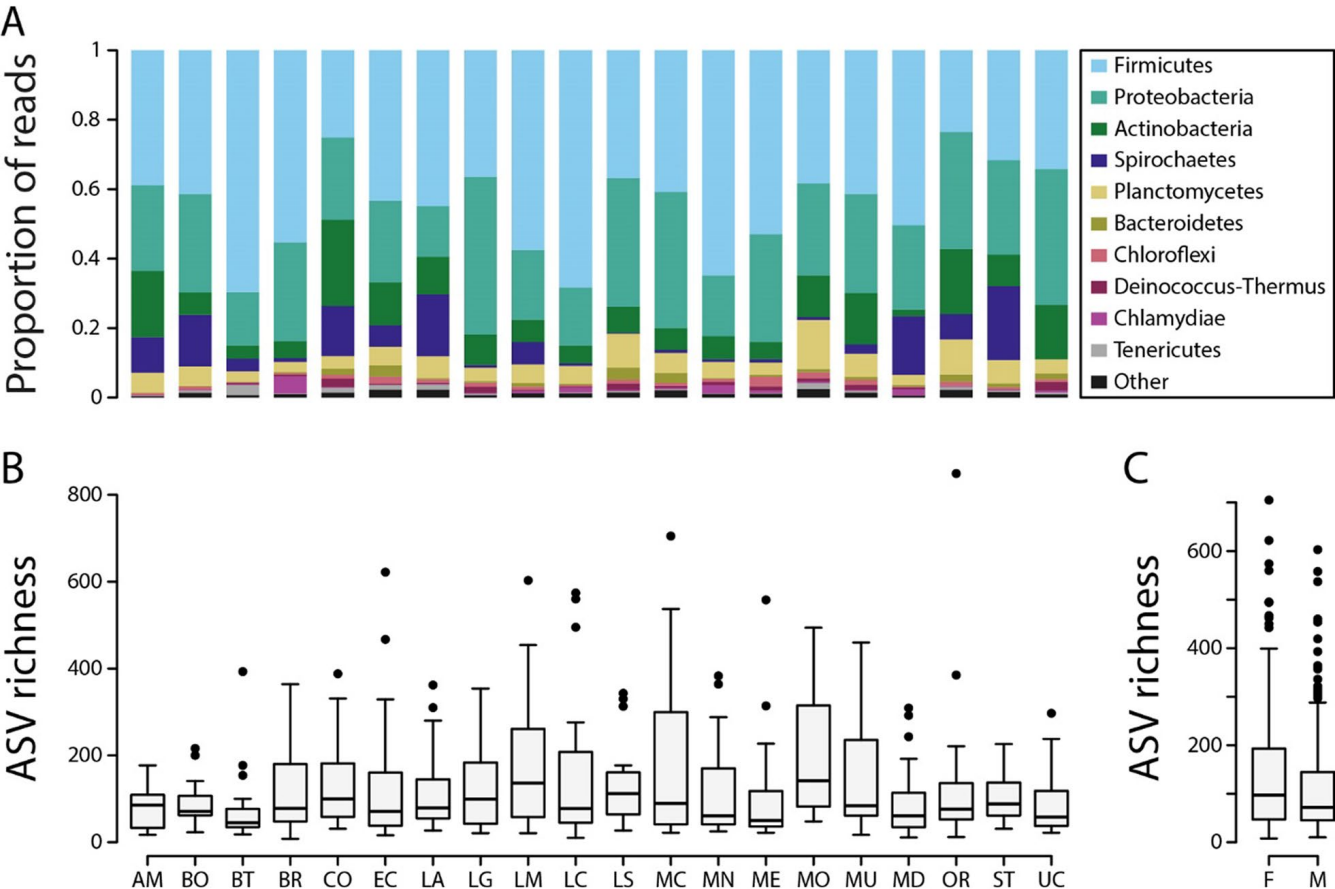
Taxonomic bar plots on the phylum level summarized by population (**A**). Alpha diversity, shown as ASV richness, by population (**B**) and by host sex (**C**)

The sequencing runs produced a total of 21,912,056 raw sequencing reads, with mean values of 31,243 reads per stickleback gut and 56,719 reads per water sample (Table S1). We imported sequence data into QIIME2 [17] and used only forward reads for our analyses, as some samples had low sequencing depth, and merging with reverse reads led to substantial read loss, likely due to lower sequence quality at the ends of the reverse reads. To maximize the number of samples retained in our analyses, we opted for higher coverage with forward reads rather than merging. Trimming parameters were determined based on visual inspection of the quality score plot. Specifically, we trimmed the first 10 base pairs of the forward reads due to low quality and truncated them after 260 base pairs because of a noticeable drop in quality scores beyond this point. This resulted in a final read length of 250 bp, which constitutes 86% of the amplified region. Briefly, we used the QIIME2 plugin *dada2* with default settings for sequence quality check, read correction, and chimera filtering in order to obtain amplicon sequencing variants (ASVs) [20]. We then constructed a bacterial phylogeny with FastTree 2.1.3 [57] and assigned taxonomy according to the SILVA 138 ribosomal RNA (rRNA) database at a 99% similarity threshold [58]. Finally, we removed chloroplasts, mitochondria, and archaea and further filtered ASVs that were found only in a single sample and had less than 10 reads or could not be assigned below the phylum level. After filtering, we retained an average of 18,611 reads per stickleback gut and 24,422 reads per water sample (Table S1). We normalized our ASV table using scaling with ranked subsampling (SRS) with a C_min_ of 2500 reads [10]. After normalization, we retained a total of 617 samples. For stickleback guts, sample sizes per lake ranged from 23 to 30, except for Amor Lake which had only 18samples. For water samples, the sample sizes ranged from 3 to 4 (Table S2). We visualized taxonomic composition of the gut microbiota for each stickleback population on the level of bacterial phyla; only phyla comprising more than 2% of the microbiota in any of the populations are shown (Fig. 2A).

We used three metrics to describe alpha diversity (bacterial diversity of individual hosts): ASV richness, Shannon diversity, and Faith’s phylogenetic diversity, and three metrics to describe beta diversity (dissimilarity of bacterial communities among hosts): Bray–Curtis dissimilarity, unweighted and weighted UniFrac [41, 42]. Across all populations, we used negative binomial generalized linear mixed-effects models (g*lmer.nb* function in the ‘lme4’ package v1.1–31) [78] for ASV richness to account for skewness and overdispersion of count data. For Shannon diversity and Faith’s phylogenetic diversity we used linear mixed-effects models (*lmer* function in the ‘lme4’ package v1.1–31). All models included lake as random effect and nine fixed effects: host sex, body length, the first three principal components obtained from geometric morphometric measurements of stickleback body shape, as well as temperature, dissolved oxygen, conductivity, and pH of the lake environment. We then used type III ANOVA to test for statistical significance of the model terms (*Anova* function in the ‘car’ package v3.1–0). We further tested for effects of host sex, body length, and the first three geometric morphometrics principal components on the three alpha diversity metrics separately for each population, thereby assessing the repeatability and variability of sex and morphology effects on gut microbiota composition. *P* values were adjusted for multiple testing using FDR correction.

To test for dissimilarity of microbial communities, we used PERMANOVA (*adonis2* function in the ‘vegan’ package v2.6-2) [3, 56]. First, we ran PERMANOVA with lake and sample type as independent variables to determine whether bacterial community composition is mainly structured geographically across lakes or between host-associated and free-living communities (fish guts vs. lake water). Next, we ran PERMANOVA with host sex, body length, the first three geometric morphometrics principal components, as well as temperature, dissolved oxygen, conductivity, and pH of the lakes as independent variables. Similar to the alpha diversity analyses, we also tested for the effects of host sex, body length, and the first three geometric morphometrics principal components on the three beta diversity metrics separately for each population to assess repeatability of observed patterns across independent populations. *P* values were adjusted for multiple testing using FDR correction. To study the core gut microbiota, we identified the ASVs shared among 50% and 80% of hosts and assigned their taxonomy on the bacterial family level. Additionally, we performed the analysis at the bacterial family level by collapsing the ASV table to family-level taxonomy and identifying the bacterial families shared among 50% and 80% of hosts. We conducted these analyses within each population and also across all populations. We further calculated within-population beta diversity for each lake to obtain information on how dissimilar hosts of the same population are. Moreover, we generated population-level distances matrices and used multiple regression on distance matrices (MRM), an extension of partial Mantel analysis, which allows assessing the relationship between a dependent distance matrix and one or more independent distance matrices. We used MRM to test for effects of divergence in free-living bacterial communities of the lakes, host morphology and genetics, as well as geographic distance on the three gut microbiota beta diversity metrics (*MRM* function in the ‘ecodist’ package v2.1.3) [26]. For this analysis, we only used a subset of 15 lakes for which we had all the data mentioned above.

## Results

The taxonomic composition of stickleback gut microbiota revealed both commonalities and variation across populations. The most abundant phylum was Firmicutes, accounting for an average of 44.8% of sequencing reads, with population averages ranging from 23.5% to 69.6%. This was followed by Proteobacteria at 27.2% (ranging from 14.6% to 45.4%), Actinobacteria at 10% (ranging from 2% to 24.8%), Spirochaetes at 6.4% (ranging from 0.1% to 21.3%), and Planctomycetes at 5.8% (ranging from 2.9% to 14.1%) (Fig. 2A). These findings are consistent with previous studies on the stickleback gut microbiota, highlighting the shared taxonomic composition across studies [16, 63, 74]. While the major bacterial phyla were shared (Fig. 2A), the observed differences in relative abundances underscore the dynamic nature of the stickleback gut microbiota across wild populations. This variation offers a unique opportunity to investigate the factors that explain the observed differences in bacterial community composition.

### Across- and within-population variation in alpha diversity

Alpha diversity of the stickleback gut microbiota exhibited considerable variation across populations. Mean alpha diversity on the population level ranged from 69.5 to 205.7 for ASV richness (Fig. 2B), from 2.5 to 4.1 for Shannon diversity and from 9.2 to 19.4 for Faith’s phylogenetic diversity. We then tested for effects of host and environmental factors on alpha diversity. Host factors included the first three principal component axes obtained from geometric morphometrics. PC axes one and three explained 25.5% and 14.8% of body shape variation, respectively, with body depth, mouth position, and pectoral fin position loading along these axes. PC axis two explained 17.5% of body shape variation and was primarily associated with caudal peduncle length (Figure S1). Host sex was associated with two alpha diversity metrics (ASV richness: *χ*^*2*^ = 11.53, *P* < 0.001; Faith’s phylogenetic diversity: *χ*^*2*^ = 10.51, *P* = 0.001) and potentially also with Shannon diversity (*χ*^*2*^ = 2.81, *P* = 0.094). Further, we found suggestive evidence for associations of the second principal component of geometric morphometric analysis with all three alpha diversity metrics (ASV richness: *χ*^*2*^ = 3.80, *P* = 0.051); Shannon diversity: *χ*^*2*^ = 3.48, *P* = 0.062; Faith’s phylogenetic diversity: *χ*^*2*^ = 2.79, *P* = 0.094), and of body length with ASV richness (*χ*^*2*^ = 2.76, *P* = 0.097) and Faith’s phylogenetic diversity (*χ*^*2*^ = 3.00, *P* = 0.083). Pairwise comparisons revealed that females had 24.4% higher ASV richness (Fig. 2C), 6.2% higher Shannon diversity and 16.4% higher Faith’s phylogenetic diversity compared to males. Yet, no significant effects of environmental parameters on any of the alpha diversity metrics were detected.

When analyzing populations separately, we found little evidence for effects of host sex and morphology on alpha diversity after FDR correction. ASV richness was only associated with host sex in Comox Lake (*χ*^*2*^ = 8.09, *P* = 0.045) and Lower Campbell Lake (*χ*^*2*^ = 9.03, *P* = 0.045) and with body length in McCreight Lake (*χ*^*2*^ = 16.67, *P* = 0.001). The second geometric morphometrics principal component was associated with ASV richness in Lower Campbell Lake (*χ*^*2*^ = 11.54, *P* = 0.014), and potentially also in Brewster Lake (*χ*^*2*^ = 6.54, *P* = 0.070) and McNair Lake (*χ*^*2*^ = 7.27, *P* = 0.070) (Table S3). No within-population effects of host sex and morphology were found for Shannon diversity (Table S4) or Faith’s phylogenetic diversity (Table S5).

### Within- and across-population core microbiota and within-population gut microbiota dissimilarity

**Fig. 3:**
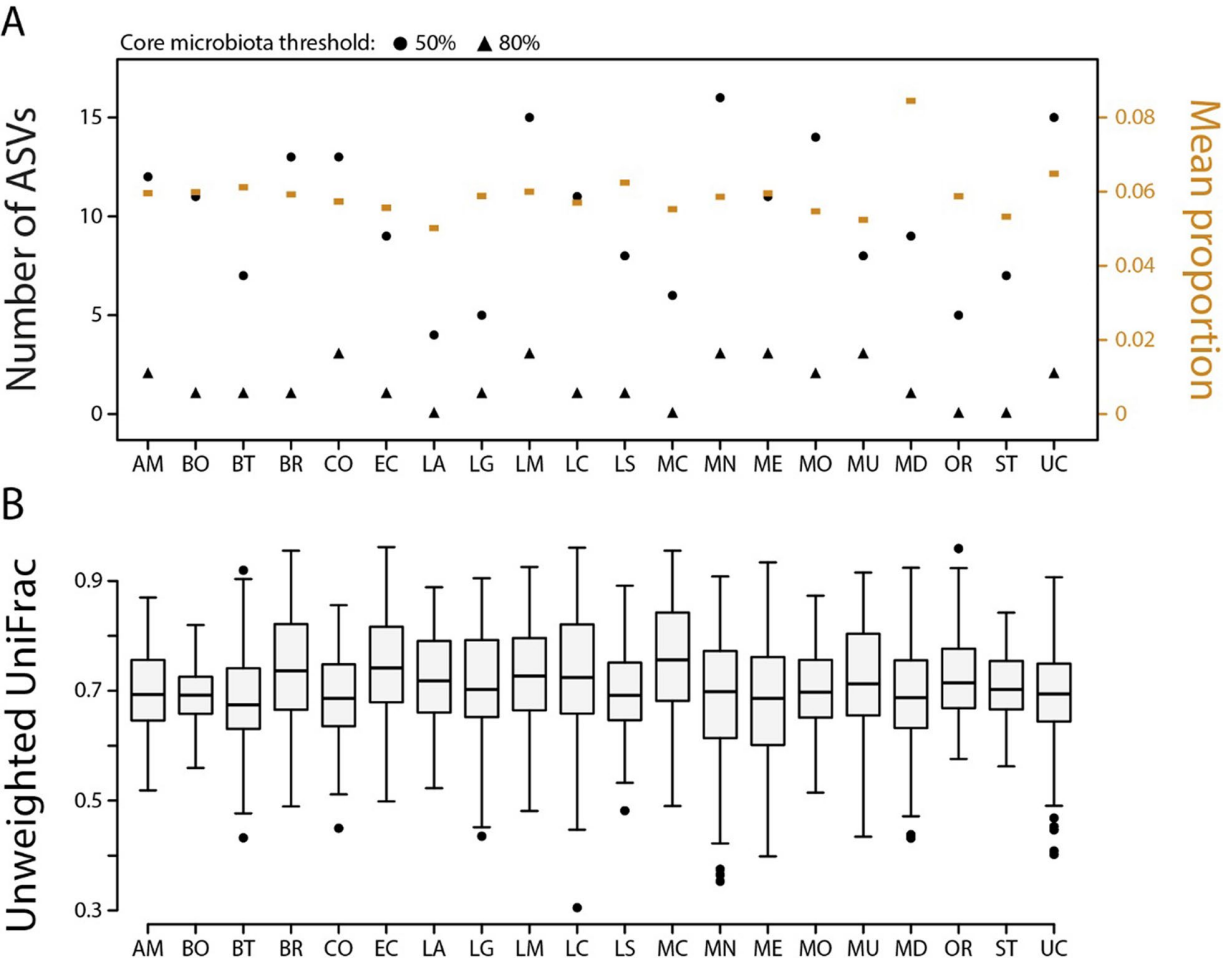
Core gut microbiota (**A**) and within-population beta diversity across stickleback populations (**B**). The number of core ASVs at the 50% threshold ranged from 4 to 16 across lakes, while at the 80% threshold, it ranged from 0 to 3 (**A**). On average, ASVs were shared among 5% to 8.5% of individuals within a population (**A**). Within-population beta diversity was consistently high across lakes based on unweighted UniFrac, with average values ranging from 0.73 to 0.76 (**B**). Together, these results demonstrate limited evidence for a core microbiota and high within-population variability across wild stickleback populations on the ASV level (see Table S6 for core bacterial families)

Across all populations, we found little evidence for a core gut microbiota. On average each ASV was shared among 3.2 hosts, which represents 0.6% of all hosts surveyed in this study. Further, a large proportion of ASVs, 13,925 out of 21,263 representing 65.5%, were found only in a single host. Only four ASVs were shared among more than 50% of all hosts; two of these ASVs belonged to the bacterial family Halomonadaceae, and the other two belonged to the Burkholderiaceae and Mycobacteriaceae. No ASV was shared among more than 80% of all hosts. When studying the core gut microbiota at the bacterial family level, we found 19 families to be shared across 50% of all hosts, including the three families mentioned above (Table S6). At the 80% threshold, only three families (Bacillaceae, Burkholderiaceae, Halomonadaceae) were part of the core gut microbiota. Next, we determined the core gut microbiota on the population level with mixed results across populations: the number of ASVs shared among 50% of hosts ranged from four in Lawson Lake to sixteen in McNair Lake whereas the number of ASVs shared among 80% of hosts ranged from zero in four lakes (Lawson, McCreight, Ormund, and Stella) to three in five lakes (Comox, Little Mud, McNair, Merrill, and Muchalat) (Fig. 3A). The number of bacterial families shared among 50% of hosts ranged from nine in Boot Lake to 32 in Mohun Lake whereas the bacterial families shared among 80% of hosts ranged from two in five lakes (Boot, Brewster, Lawson, Lower Campbell, and McCreight) to 12 in Mohun Lake (Table S6).

At the 50% threshold we found a total of 50 core ASVs on the population level; 22 of them were part of the core microbiota only in one of the populations. Yet, six ASVS were part of the core microbiota in more than ten populations and two of these each belonged to the bacterial families Burkholderiaceae and Halomonadaceae and one each belonged to the Bacillaceae and Mycobacteriaceae. Notably, one of the Halomonadaceae ASVs was part of the core microbiota in all twenty populations. At the 80% threshold, we only found eleven core ASVs on the population level; nine of which were part of the core in one or two populations. Only two ASVs were part of the core microbiota in seven populations and these ASVS belonged to the families Burkholderiaceae and Halomonadaceae. Moreover, there was little variation in the number of hosts each ASV was shared among with average values ranging from 1.5 to 1.8 individuals, representing 5% to 8.5% of the populations (Fig. 3A). This weak evidence for a core microbiota on the population level based on ASVs was corroborated by relatively high levels of within-population beta diversity across all lakes (Fig. 3B). Based on the three beta diversity metrics, values ranged from 0.92 to 0.95 (Bray–Curtis dissimilarity), 0.73–0.76 (unweighted UniFrac), and 0.55–0.67 (weighted UniFrac). In sum, most ASVs were found only in one or very few hosts and only a small number of ASVs was found in the majority of hosts within each population whereas higher rates of sharing were observed on the bacterial family level.

### Gut microbiota dissimilarity on the individual host level (within- and across-population beta diversity)

**Fig. 4:**
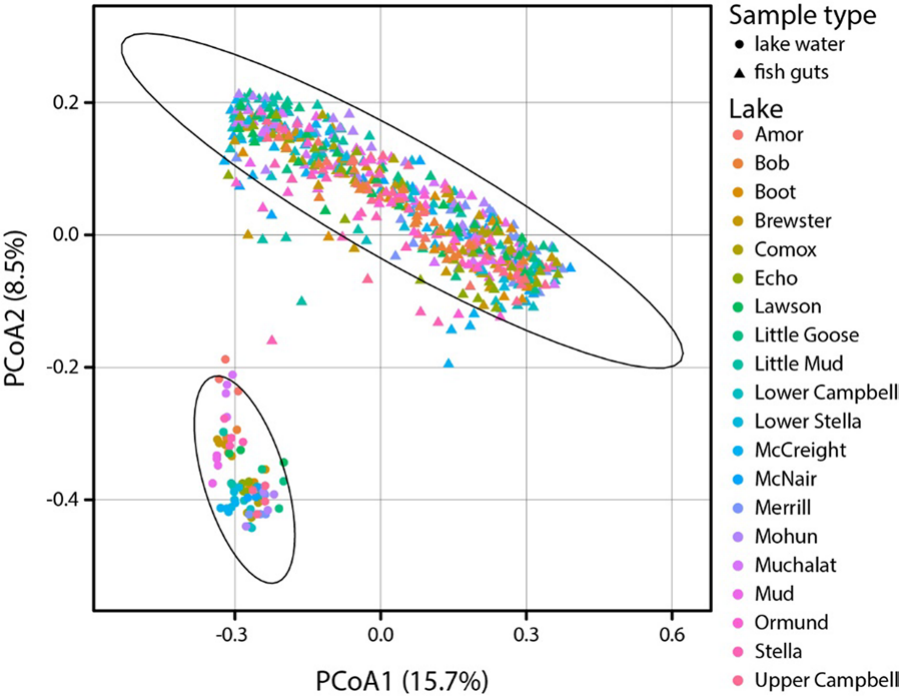
Principal Coordinates Analysis (PCoA) plot based on the unweighted UniFrac metric illustrating the variation in microbial community composition among fish guts (triangles) and water samples (circles) collected from different lakes. Each point represents a sample with colors indicating the lake of origin. The axes correspond to the first two principal coordinates, which capture the majority of the variance in the data. Ellipses denote the 99% confidence intervals for the clustering of sample types (fish guts and lake water)

We found that microbial communities from stickleback guts and lake water differed substantially (Bray–Curtis: *R*^*2*^ = 0.071, *F* = 51.174, *P* = 0.001; unweighted UniFrac: *R*^*2*^ = 0.097, *F* = 70.185, *P* = 0.001; weighted UniFrac: *R*^*2*^ = 0.132, *F* = 102.489, *P* = 0.001) (Fig. 4). Focusing specifically on the gut microbiota, we found that host population explained a substantial proportion of gut microbiota dissimilarity across three beta diversity metrics (Bray–Curtis: *R*^*2*^ = 0.125, *F* = 3.928, *P* = 0.001; unweighted UniFrac: *R*^*2*^ = 0.095, *F* = 2.886, *P* = 0.001; weighted UniFrac: *R*^*2*^ = 0.137, *F* = 4.365, *P* = 0.001). To obtain more detailed insights, we then determined the main contributors to bacterial community dissimilarity across stickleback populations by testing host-associated (sex, body length, morphology) and environmental (temperature, dissolved oxygen, pH) factors. While all the tested factors affected gut microbiota dissimilarity (except for dissolved oxygen on weighted UniFrac), none of them explained more than 1% of the variation in beta diversity (except for temperature which explained 1.2% of the variation based on weighted UniFrac). All these factors combined explained 4.1–6.7% of the variation in beta diversity based on the three metrics (Table S7).

When testing beta diversity among individuals of each population separately, we obtained similar results as for alpha diversity: only in a few populations did we find evidence for associations between host sex or morphology and beta diversity after FDR correction. We only found effects of host sex (*R*^*2*^ = 0.113, *F* = 2.713, *P* = 0.04) and potentially of the third geometric morphometrics principal component (*R*^*2*^ = 0.105, *F* = 2.525, *P* = 0.08) in Bob Lake based on Bray–Curtis dissimilarity (Table S8). There was no evidence for effects of host sex or morphology on unweighted UniFrac (Table S9) or weighted UniFrac (Table S10) in any population. Next, we tested for correlations between beta diversity and morphological divergence among fish from the same lake. However, we found no evidence for such correlations in any of the populations and any of the three beta diversity metrics (Tables S11–13).

### Gut microbiota dissimilarity on the population level (across population beta diversity)

**Fig. 5:**
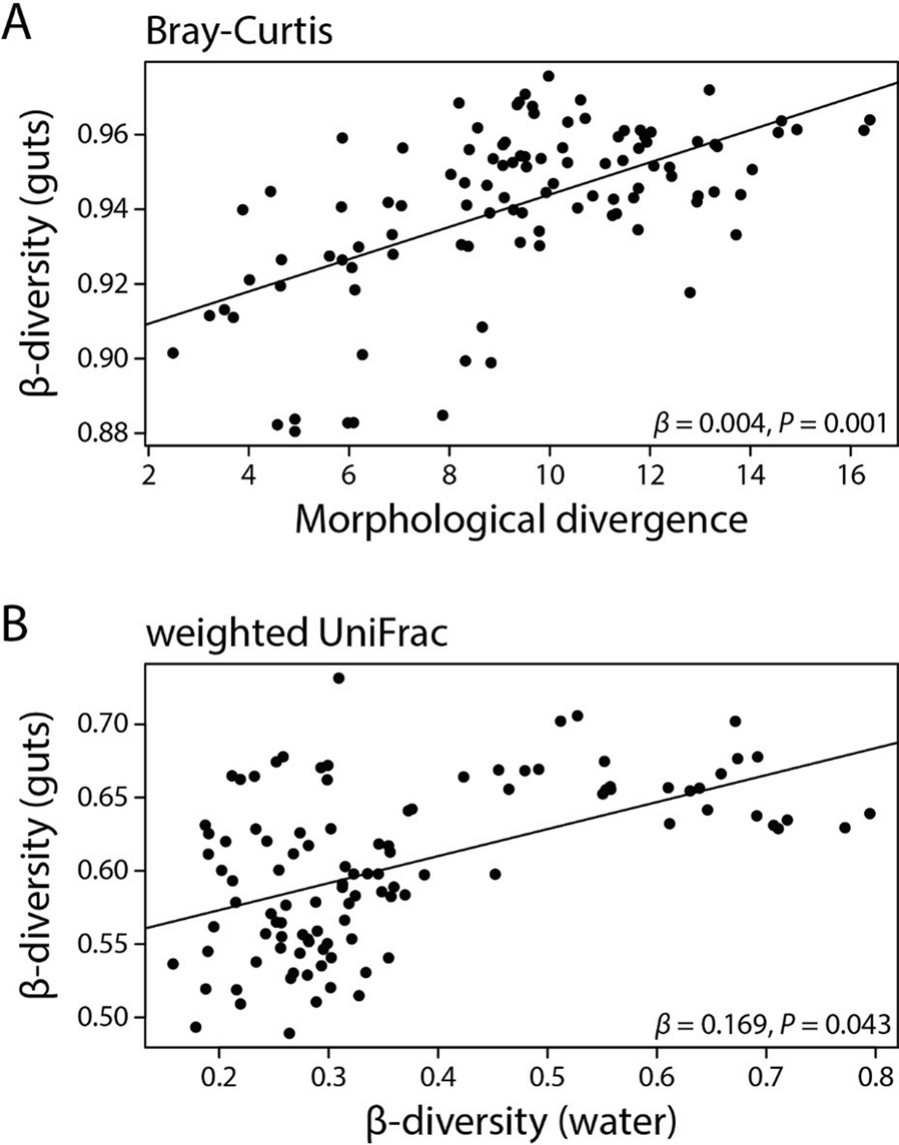
We used multiple regression on distance matrices (MRM) to find whether gut microbiota divergence (beta diversity) on the population is associated with divergence in free-living bacterial communities of the lakes, host morphology and genetics, as well as geographic distance. Each data point represents a pairwise comparison between two populations for gut microbiota beta diversity and stickleback morphology and between two lakes for lake water beta diversity. Population-level beta diversity (based on Bray–Curtis dissimilarity) was explained by host morphological divergence (**A**) and by divergence in free-living bacterial communities (based on weighted UniFrac) (**B**). No significant predictors were found for unweighted UniFrac

Next, we used multiple regression on distance matrices (MRM) to test the effects of divergence in free-living bacterial communities of the lakes, host morphology and genetics, and geographic distance on the three gut microbiota beta diversity metrics summarized on the population level. For this analysis, we selected a subset of 15 lakes for which all the data mentioned above was available. For Bray–Curtis dissimilarity, the model was significant (*F* = 12.379, *P* = 0.001) and explained 38.5% of the variation in gut microbiota beta diversity; host morphological divergence, determined through geometric morphometrics and across-population Mahalanobis distances, was the only significant predictor (*β* = 0.004, *P* = 0.001) (Fig. 5A). For unweighted UniFrac, the model was not significant (*F* = 0.645, *P* = 0.947) and only explained 3.2% of the variation with no significant predictor variables. For weighted UniFrac, the model was significant (*F* = 11.437, *P* = 0.032) and explained 36.6% of the variation with divergence in free-living bacterial communities as the sole significant predictor (*β* = 0.169, *P* = 0.043) (Fig. 5B).

## Discussion

This study examined how host morphology, biological sex, genetic divergence, and environmental factors shape gut microbiota composition and diversity in wild threespine stickleback from 20 lakes on Vancouver Island, Canada. Across populations, we found substantial variation in taxonomic composition and diversity of gut microbial communities (Figs. 2, 4). Alpha diversity was affected by host sex (Fig. 2C) and morphology (body shape), but not by environmental characteristics, suggesting a stronger effect of host traits compared to the lake environment. Beta diversity was affected by both host traits and environmental characteristics but each factor explained only a very small proportion (< 1%) of its variation. Multiple regression on distance matrices across populations revealed that gut microbiota divergence among stickleback populations (based on Bray–Curtis dissimilarity) is associated with morphological divergence and with divergence of environmental bacterial communities (based on weighted UniFrac). Yet, within-population analyses found limited evidence for associations between host traits and gut microbiota alpha and beta diversity, with only a few significant effects. Overall, our study hints at the relative importance of host traits over environmental characteristics in shaping the stickleback gut microbiota, with implications for understanding host-microbiota interactions in natural environments.

The core microbiota consists of microbial taxa consistently associated with a host or environment, though definitions vary [54, 71]. Here, we defined core ASVs or families as those present in at least 50% or 80% of host individuals. Based on ASVs, our results indicate a weakly defined core gut microbiota, both within and across stickleback populations, with limited ASV sharing among individuals and high within-population beta diversity (Fig. 3B). No population exhibited a strongly defined core (Fig. 3A), highlighting the individuality of gut microbial communities and underscoring the complexity of microbial colonization even among fish within the same population. However, core ASVs found across multiple populations belonged to a few bacterial families (Halomonadaceae, Burkholderiaceae, Mycobacteriaceae), suggesting potential functional importance. While these families were identified as part of the core microbiota, the representation is based on one or two ASVs each which limits the robustness of any conclusions that can be made. Yet, this finding was supported by the observation that these same families were consistently part of the core microbiota when described at the bacterial family level. The genus Burkholderia has been found to be part of the core microbiota of lake whitefish [70] and zebrafish [64], hinting at the importance of these bacteria for their fish hosts. It is currently unclear how our results compare to other fish (or vertebrate) species, as measures and thresholds to define the core microbiota are inconsistent across studies (reviewed in [54]). A comprehensive meta-analysis, as has been done for reptiles [35], would help clarify how core microbiota structure varies across hosts and environments.

One key observation from our study is the substantial influence of host population on gut microbiota composition, suggesting that ecological differences among stickleback populations and their respective environments may shape the taxonomic composition and diversity of gut microbial communities, potentially through processes such as ecological filtering due to differences in the abiotic conditions of the gut environment—such as pH, diet, or microbial exposure. Previous work in stickleback found support for host diet [15, 31, 63, 74] and genotype [73–75] being important contributors to gut microbiota variation within and among populations. In our study, gut microbiota divergence among populations appeared to be associated with overall host morphological divergence (Fig. 5A), but not with genetic divergence. In stickleback, body shape variation is strongly linked to trophic ecology, particularly the well-documented benthic-limnetic divergence [6, 68]. Benthic stickleback, which inhabit structurally complex, vegetation-rich environments, typically have deeper bodies and downturned mouths adapted for bottom-feeding. In contrast, limnetic stickleback, which occupy open-water habitats, exhibit more streamlined bodies and terminal mouths suited for zooplankton feeding [6, 47]. In our geometric morphometric analysis, PC1 and PC3 primarily captured variation in body depth, a trait distinguishing benthic (deeper-bodied) and limnetic (more streamlined) ecotypes and mouth position, aligning with the benthic-limnetic trophic differences (Figure S1) [6, 68]. However, PC2 did not clearly correspond to benthic-limnetic divergence, suggesting it may capture variation unrelated to trophic ecology. While our results suggest a potential link between body shape and gut microbiota composition, further research is needed to establish whether this is related to trophic niche differences. To gain more detailed insights into the relationship between host morphology, trophic ecology, and gut microbiota composition, investigating traits that directly impact feeding success, such as gill raker number and length, will be instrumental [68]. In order to draw more robust conclusions, diet information would also need to be collected for individual hosts using stable isotope analysis or metabarcoding of gut contents [55].

The lack of an association between genetic divergence and gut microbiota divergence was rather surprising but could be driven by overall low levels of genetic divergence among the populations included in our study (F_ST_: 0.026–0.091). In contrast, a study on Oregon stickleback found an effect of genetic divergence on gut microbiota, but F_ST_ values were much higher (0.021–0.575; [75]). Thus, we hypothesize that the limited genetic divergence among our populations was insufficient to structure gut microbiota composition. Our findings suggest that, at least at the genome-wide level, gut microbiota variation in wild stickleback is more likely structured by host traits (e.g., morphology, trophic ecology, sex) and environmental factors rather than by broad genetic differentiation among populations. Although our genetic divergence estimates were obtained from a previous study [12] and come from different individuals than those used for gut microbiota analysis, genome-wide allele frequencies are unlikely to shift substantially across years, making it unlikely that this methodological limitation influenced our results. Notably, despite low genetic divergence, we observed high beta diversity and gut microbiota structuring across populations, reinforcing the role of ecological factors. Prior work has shown that ecological divergence can arise with minimal genomic divergence [12, 28]. Additionally, our study links morphology to gut microbiota diversity (Fig. 5A), suggesting that body shape may reflect ecological adaptations influencing microbial community composition. The association between divergence in free-living bacterial communities and gut microbiota divergence (based on weighted UniFrac) further supports the role of environmental microbial communities in shaping host-associated microbiota.

We detected mixed evidence for potential effects of host traits and environmental characteristics on gut microbiota alpha and beta diversity across stickleback populations. Host sex explained small but significant variation in beta diversity and females consistently had higher alpha diversity across all metrics (Fig. 2C). Evidence for sex-dependent differences in gut microbiota composition and diversity is inconsistent across species. Several studies in mammals found no or negligible effects of host sex on the gut microbiota of humans [39], ring-tailed lemurs [7], mice [38], and North American red squirrels [11, 61]. In contrast, there are sex-specific gut microbiota differences in chimpanzees which might be produced by differences in feeding behavior [22]. In stickleback, previous studies found sex-dependent diet effects within a single population [16]. However, no consistent sex-based differences were found across two populations and their hybrids [73], ten populations from lakes, rivers and estuaries [74] or in benthic and limnetic ecotypes reared in experimental ponds [29]. Thus, our study is the first to provide compelling evidence for sex-dependent differences in gut microbiota alpha and beta diversity across wild stickleback populations.

Notably, there was an association between divergence in gut microbial and free-living bacterial communities based on the weighted UniFrac metric (Fig. 5B), suggesting some influence of the lake bacterial communities on the stickleback gut microbiota. Yet, gut and water samples clustered distinctly (Fig. 4), consistent with previous studies showing that gut microbial communities of fish are often highly distinct from their free-living counterparts of the aquatic environment [33, 67, 70]. Environmental microbes can enter the gut via water and food where they may pass through, fail to establish, or become part of the resident microbiota. Indeed, a previous study in stickleback showed that many gut bacteria may be acquired from the aquatic environment [74], underscoring its role in microbiota assembly. It remains to be determined whether the observed association between gut-associated and free-living bacterial communities results from direct microbial uptake or from shared environmental conditions in the lake (e.g., nutrient availability, temperature, or microbial interactions) shaping both communities in similar ways. However, geographic distance between lakes did not influence gut microbiota composition, making it unlikely that spatially structured environmental variation drives this correlation. If environmental gradients were to shape both free-living and host-associated bacterial communities, a higher similarity of environmental conditions (e.g., temperature and precipitation) might be expected between lakes in close proximity. A decrease in compositional similarity among bacterial communities with geographic distance (i.e., distance-decay of similarity) [53] has been found in free-living bacterial communities from various habitats and geographic regions [21, 44, 45]. Yet, our results suggest that distance-decay of similarity might not apply to host-associated gut microbial communities. In sum, while the stickleback gut microbiota is distinct from free-living bacterial communities, environmental bacteria appear to play a significant role in shaping its composition, highlighting the complex interactions between host, gut, and environment.

While several host traits and environmental characteristics had small but significant effects on gut microbial communities across populations, within-population analyses revealed much weaker patterns. Significant associations between host sex or morphology and gut microbiota were observed in only one or two lakes per metric, suggesting inconsistency across populations. Regarding host sex, it remains unclear why some populations show gut microbiota differences between males and females while others do not. This may be due to population-specific differences in sexual dimorphism of diet, trophic traits or physiology [16, 48, 60]. Lower sample sizes within lakes and variation in data dispersion across populations might further contribute to inconsistent patterns. Importantly, population-level differences in gut microbiota composition were much larger than differences associated with host traits, indicating that the gut microbiota is primarily structured by population, rather than individual host traits.

## Conclusions

Our study underscores the multifaceted nature of host-microbiota interactions and the importance of considering both host-associated and environmental factors in unraveling gut microbiota diversity across wild host populations. Notably, we found strong divergence in gut microbiota composition among closely related populations that show minimal genetic divergence [28] but substantial variation in trophic ecology along the benthic-limnetic axis [12]. This finding suggests that dietary differences may drive substantial microbiota differentiation across populations, though the role of other environmental factors, such as local abiotic conditions and biotic interactions, and host-parasite interactions, warrants further investigation. Future research would benefit from a more integrated approach that combines morphological, dietary, and genetic analyses with gut microbiota characterizations within the same host individuals. This could help to deepen our understanding of the mechanistic underpinning of these interactions. Despite these limitations, our findings contribute to the growing body of knowledge on microbial ecology and have implications for our understanding of the eco-evolutionary dynamics of host-associated microbial communities. By providing evidence for the role of trophic ecology in shaping microbiota divergence, this study reinforces the importance of exploring eco-evolutionary dynamics of host-microbiome interactions across diverse ecological contexts.

## Supplementary Information

Supplementary material 1

Supplementary material 2

## Data Availability

All data files and R scripts (https://figshare.com/s/3a737e8e305dba53d080) and the raw sequencing reads from fish collected in 2020 (https://figshare.com/s/29ce7952f18375a6ad5a), 2021 (https://figshare.com/s/40186de9b83dec7270a6), 2022 (https://figshare.com/s/b4f67fabf1c88e1926d4) and from environmental DNA obtained from lake water (https://figshare.com/s/841fafffd278ed26a197) have been deposited on figshare.
