# Supplementary material for: Host traits and environmental variation shape gut microbiota diversity in wild threespine stickleback
Source: Anim Microbiome. 2025 Jun 18;7:67. doi: 10.1186/s42523-025-00404-0 (PMC12177961; doi:10.1186/s42523-025-00404-0)
Supplement: Supplementary file 1 — Supplementary material 1 [file 42523_2025_404_MOESM1_ESM.pdf]

Supporting Information for “Host traits and environmental variation shape gut microbiota diversity in wild threespine stickleback”

Andreas Härer (AH)[1]

Emma Kurstjens (EK)[1,2]

Diana J. Rennison (DJR)[1]

[1] School of Biological Sciences, Department of Ecology, Behavior, & Evolution, University of California San Diego, La Jolla, California, USA

[2] Department of Medicine, School of Medicine, University of California San Diego, La Jolla, California, USA

Supplementary Figures

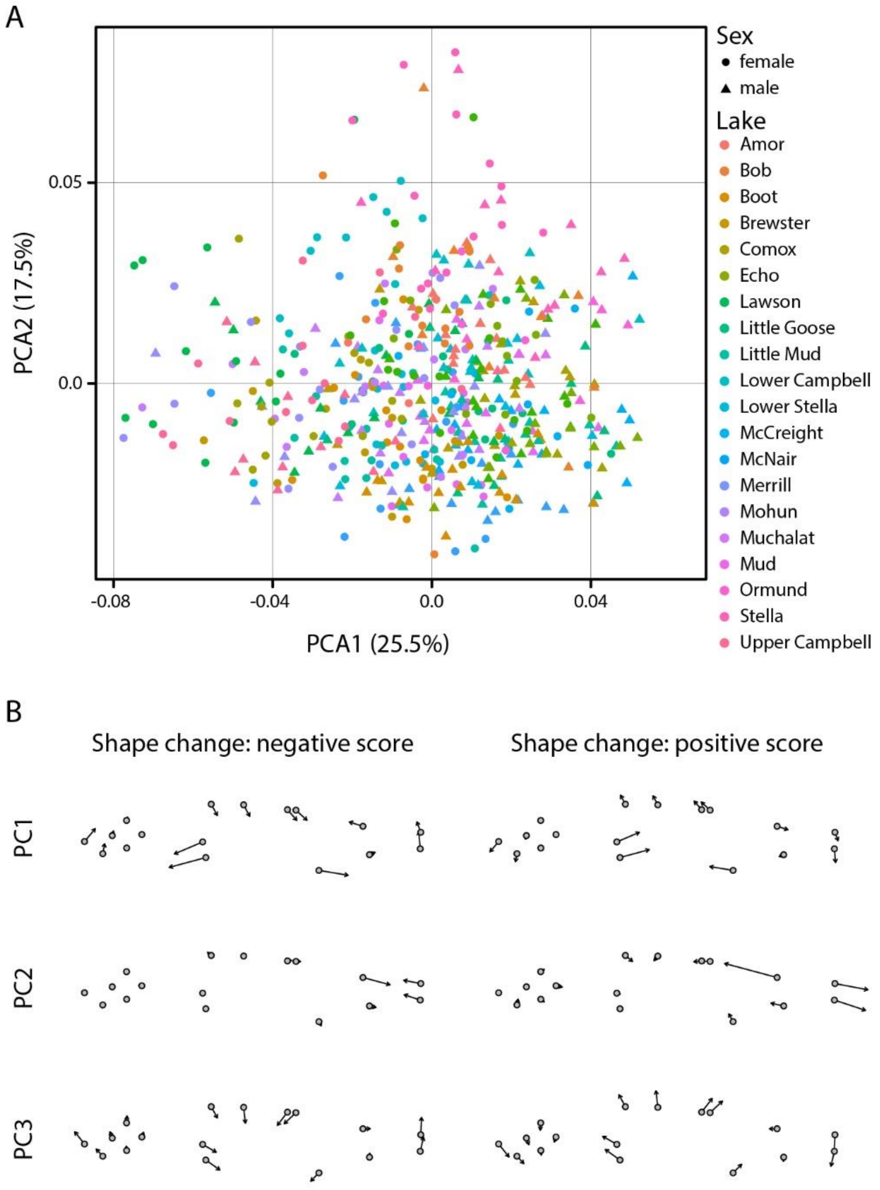

Figure S1: Body shape variation based on geometric morphometrics. Scatterplot of principal component (PC) scores along the first two PC, with data points colored by population (A). Circles represent female fish, and triangles represent male fish. Visualization of body shape changes associated with the most negative (left) and most positive (right) PC scores for PC1, PC2, and PC3 based on 17 landmarks (B). Arrows illustrate the major axes of body shape variation captured by each principal component.
